# Study of Interdigitated Electrode Arrays Using Experiments and Finite Element Models for the Evaluation of Sterilization Processes

**DOI:** 10.3390/s151026115

**Published:** 2015-10-14

**Authors:** Jan Oberländer, Zaid B. Jildeh, Patrick Kirchner, Luisa Wendeler, Alexander Bromm, Heiko Iken, Patrick Wagner, Michael Keusgen, Michael J. Schöning

**Affiliations:** 1 Institute of Nano- and Biotechnologies (INB), FH Aachen University of Applied Sciences, Heinrich-Mussmann-Str. 1, Jülich 52428, Germany; E-Mails: oberlaender@fh-aachen.de (J.O.); Zaid.Jildeh@imagine.de (Z.B.J.); kirchner@fh-aachen.de (P.K.); luisa.wendeler@alumni.fh-aachen.de (L.W.); alexander.bromm@alumni.fh-aachen.de (A.B.); iken@fh-aachen.de (H.I.); 2 Peter-Grünberg Institute (PGI-8), Forschungszentrum Jülich GmbH, Jülich 52428, Germany; 3 Soft Matter and Biophysics, Catholic University Leuven, Celestijnenlaan 200 D, Leuven 3001, Belgium; E-Mail: patrick.wagner@fys.kuleuven.be; 4 Institute of Pharmaceutical Chemistry, Philipps-University Marburg, Marbacher Weg 6-10, Marburg 35037, Germany; E-Mail: keusgen@staff.uni-marburg.de

**Keywords:** sterilization process, hydrogen peroxide, interdigitated electrodes (IDE), FEM model

## Abstract

In this work, a sensor to evaluate sterilization processes with hydrogen peroxide vapor has been characterized. Experimental, analytical and numerical methods were applied to evaluate and study the sensor behavior. The sensor set-up is based on planar interdigitated electrodes. The interdigitated electrode structure consists of 614 electrode fingers spanning over a total sensing area of 20 mm^2^. Sensor measurements were conducted with and without microbiological spores as well as after an industrial sterilization protocol. The measurements were verified using an analytical expression based on a first-order elliptical integral. A model based on the finite element method with periodic boundary conditions in two dimensions was developed and utilized to validate the experimental findings.

## 1. Introduction

Sterilization processes are important in various industrial fields such as pharmaceutics, medicine and food packaging. The processes have to ensure the inactivation of all microorganisms even of highly resistant bacterial spores to achieve an extended shelf-life of products and to improve consumer safety. In food packaging industry various sterilization processes are used and the applied method depends on the object to be sterilized. For the sterilization of multi-layered composite food packages, which will contain the pre-sterilized and sensitive food product (e.g., milk and juice), hydrogen peroxide (H_2_O_2_) vapor has become the preferred choice among other chemical sterilizations [1].

H_2_O_2_ possesses strong oxidizing properties and is able to form radicals as reaction intermediates, in particular at elevated temperature. The formed radicals are chemically instable, yet they impart the microbicidal and sporicidal characteristics of H_2_O_2_. Finally, H_2_O_2_ dissociates to the environmentally friendly end-products oxygen and water vapor [2,3,4].

Prior to product filling, pre-formed food packages are guided through the sterilization chamber of the aseptic filling machine. An evaporator unit is used to form the sterilization medium at temperatures up to 300 °C from a mixture of an aqueous H_2_O_2_ solution (35% *w*/*w*) and air. The vapor mixture is then injected into the packages interacting and destroying the microorganisms present on their surface. The standard methods to evaluate the sterilization process effectiveness are microbiological challenge tests (end-point or count-reduction tests) [5,6]. For these methods the surfaces of multiple test packages are inoculated with a refined sample of a resilient microorganism. Typically, spores of *Bacillus atrophaeus* *(B. atrophaeus)* are applied as an indicator microorganism to evaluate H_2_O_2_ vapor sterilization processes. However, the mentioned testing methods require fitted laboratories, trained staff and sample preparation; therefore, these methods are expensive and time-consuming.

In this article, a faster technique to evaluate sterilization processes with H_2_O_2_ vapor is introduced. The monitoring method is based on an impedimetric analysis of *B. atrophaeus* spores before and after the sterilization process. A sensor based on an interdigitated electrode (IDE) array has been developed to enable such analysis [7]. Instead of inoculating the surface of test packages, the indicator microorganisms are immobilized directly on the sensor surface. An alternating voltage applied to the sensor terminals induces a current depending on the intrinsic properties of the sensor and the spores on its surface. By measuring and noting the relation between voltage and current, the impedance of the sensor can be calculated. Moreover, the capacitance of the sensor can also be derived using an equivalent circuit approach.

To verify and validate the sensor results, analytical expressions and numerical models were applied. For the verification of interdigitated electrode structures, an analytical expression described in earlier literature was used [8,9,10]. The sensor validation was achieved by developing a representative numerical model based on the finite element method (FEM).

## 2. Experimental Section

### 2.1. Sensor Design and Fabrication

The IDEs are designed to record transformations of the indicator microorganisms (spores of *B. atrophaeus,* DSMZ 675) induced by the sterilization process. The IDEs (as stated in [7]) consist of 614 electrodes with a thickness of 110 nm, and 5 μm width and interspacing. This geometry is imposed by the resolution limit of the applied in-house lithography process. The length of the electrodes is 3.25 mm, providing a total sensing area of about 20 mm^2^. A wall structure with a height of 400 μm was designed around the IDEs to hold a liquid sample of an ethanol-based spore suspension with a volume of 10 μL. This volume corresponds to the volume of the above mentioned challenge tests [6].

**Figure 1:**
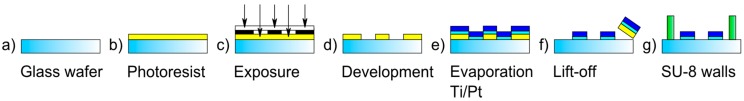
Schematic representation demonstrating the individual sensor fabrication steps.

**Figure 2:**
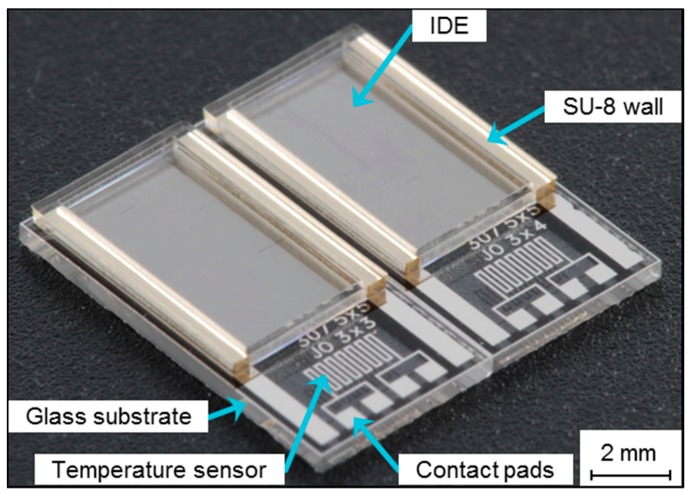
Micrograph of the sensor based on a glass substrate, depicting the IDE structure surrounded by SU-8 walls; the lower part shows the temperature sensors and contact pads.

A schematic representation of the sensor fabrication is shown in Figure 1. The sensor fabrication starts with a glass wafer (Borofloat^®^ 33, Schott GmbH, Jena, Germany), which serves as a non-conductive substrate to reduce the total parasitic capacitance [11]. The first fabrication step is wafer cleaning with acetone, isopropyl and deionized (DI) water. As adhesion promoter for the photoresist layer TI-Prime (Microchemicals GmbH, Ulm, Germany) is deposited by spin-coating at 4000 rpm. Subsequently, the photoresist AZ5214E (AZ Electronic Materials GmbH, Wiesbaden, Germany), required for the later lift-off process, is deposited by an additional spin-coating step, resulting in a final thickness of 1.4 μm. The parameters are described in detail in [7]. Hence, the electrode and meander structures are patterned by a lithography step that is performed on a mask aligner (Süss Microtec AG, Garching, Germany) using a custom-made glass mask. The meander structures were introduced at this initial stage of sensor development to set up a platform for temperature sensing. Development of the photoresist is performed in developer AZ 326 MIF obtaining the patterned surface. On top of the resulting surface two layers of titanium and platinum are deposited by electron-beam evaporation (Univex, Leybold GmbH, Cologne, Germany). The titanium layer with a thickness of 10 nm serves as an adhesion promoter between the glass substrate and the platinum electrode structure with 100 nm thickness. The final electrode structure was obtained after a lift-off process in dimethyl sulfoxide (DMSO), supported by an ultrasonic bath. For the fabrication of the wall structure a high-viscose negative-tone photoresist (SU-8 2150, Microchem, Inc., Newton, MA, USA) is deposited. A consecutive two-step prebake (7 min at 65 °C and 120 min at 95 °C) is performed to release the thermal stress of the photoresist. The resulting photoresist layer is then patterned lithographically. Prior to development, a consecutive two-step post-exposure bake (5 min at 60 °C and 30 min at 95 °C) is conducted. The developer mr-DEV 600 is applied for photoresist development and the hard bake of the wall structures was conducted at 150 °C for 15 min. The final wafer is diced into single chips of 5 × 10 mm^2^. An image of the sensor is depicted in Figure 2.

### 2.2. Impedimetric Analysis

The data acquisition was performed with an E4980A impedance analyzer (Agilent Technologies Inc., Santa Clara, CA, USA). A four terminal point-probe station is used to connect the IDEs, thus eliminating the influence of connection lines’ impedance on the measurement. An alternating voltage source of 20 mV with 0 V bias is used to excite the IDEs. While the IDEs are exposed to air, the impedance spectra are recorded in the frequency range of 500 Hz to 1 MHz with a logarithmic-scaled step size. A minimum set of two different IDEs is used to evaluate the impact of the sterilization process. One IDE serves as a reference to monitor the impact of the sterilization process on the material and the sensor structure itself. On the second IDE, spores of the indicator microorganisms (*B. atrophaeus*) are immobilized. The spore immobilization is adapted from sterility tests of aseptic packaging machines in food industry [6]. The sensing structure is drop-coated with 10 μL of an ethanol (70% *v*/*v*)-based spore suspension, which contains 10^8^ colony-forming units per milliliter (cfu/mL). This results in a spore load count of 10^6^ cfu on the sensing surface. The impedance spectrum of the IDE with immobilized spores is recorded after the complete evaporation of the ethanol solution. Measurements with the IDEs are performed at different states (blank IDE, after spore immobilization and after the sterilization process) to track changes in the impedance value.

### 2.3. Sterilization Process

The sterilization of the IDEs (reference and sensing part) is conducted in a specially designed sterilization test rig as described in [12]. This test rig is a replica of the sterilization units found in industrial aseptic food packaging machines. A defined mixture of air and an aqueous solution of H_2_O_2_ (35% *w*/*w*) is vaporized to achieve a vapor concentration of 7.5% *v*/*v* with a temperature of 240 °C at the outlet of the evaporator. The IDEs are placed for an exposure time of 0.3 s with a distance of 5.5 cm below the outlet nozzle, which provides a directed stream of H_2_O_2_ vapor. The parameters for the chosen sterilization process guarantee full sterility as shown in previous work in which microbiological sterility tests were performed in parallel to sensor-based experiments [13].

## 3. Sensor Verification and Validation

### 3.1. Sensor Verification

**Figure 3:**
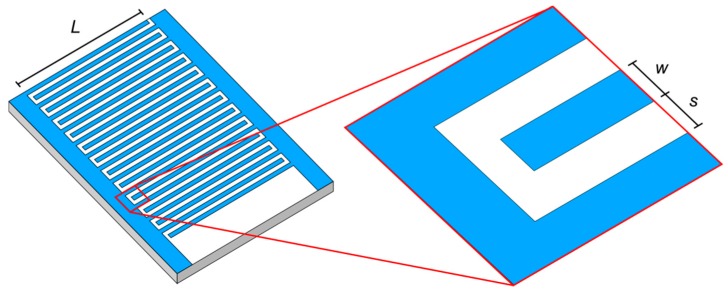
Simplified view: defining the geometric parameters of the IDE structure.

In addition to sensor measurements, a tool was required to verify the functionality of the sensor. It should give a quantitative and qualitative representation of the expected results. Therefore, an analytical expression was selected, which was proposed by Olthuis *et al.* [14] and successfully applied by several other authors [8,9]. Abu-Abed and Lindquist extended the original analytical expression to include also the capacitance contribution due to the transverse field in the narrow region between the electrode fingers [15]. Based on this prior work, the final analytical expression used to derive the capacitance of a periodic IDE sensor structure is given in Equation (1):

(1) $$C=L(N-1)\left(\frac{{\text{ε}}_{0}{\text{ε}}_{r,t}}{2}\frac{K\left({\left(1-k^{2}\right)}^{1/2}\right)}{K(k)}+2{\text{ε}}_{0}{\text{ε}}_{r,m}\frac{t}{s}\right)$$

where *C* is the calculated capacitance of the IDE structure. *L* is the length of the electrode fingers and *N* the number of unit cells. ε_0_ represents the permittivity of vacuum 8.851 × 10^−12^ As/Vm and *ε_r,t_* is the total relative permittivity surrounding the electrodes (empty sensor: air and glass; sensor with spores: porous spore layer and glass). *K*(*k*) represents the elliptical integral of first order to calculate the impact of the fringing field. The modulus *k* defined in Equation (2) is determined by the periodic structure of the electrode geometry. ε_r,m_ describes the relative permittivity present between the electrodes to represent the capacitance formed due to the transverse field (empty sensor: air; sensor with spores: porous spore layer). The geometrical parameters *t*, *s*, and *w* are the electrode thickness, interspacing between electrodes and the width of electrode fingers, respectively (see Figure 3):

(2) $$k=\cos\left(\frac{\pi }{2}\frac{w}{s+w}\right)$$

### 3.2. Sensor Validation

**Figure 4:**
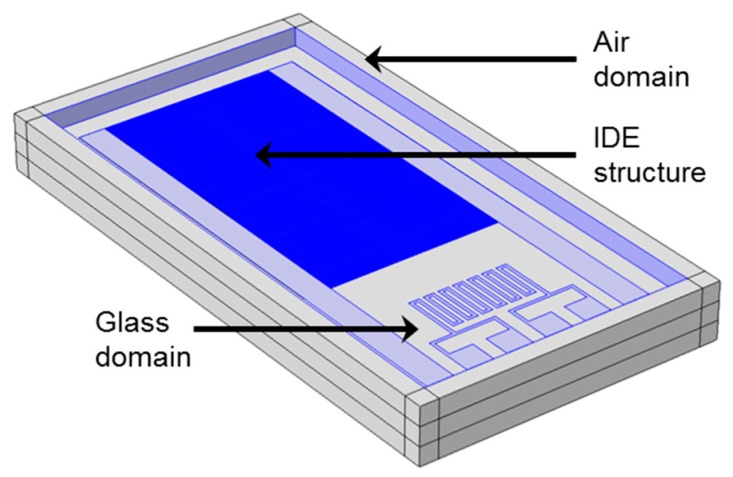
FEM-generated 3D sensor model including the IDE structure surrounded by air and glass domains.

The initial idea was to model and simulate the full 3D geometry of the sensor and its surroundings (air and sensor substrate) as shown Figure 4. The geometrical ratio between the thickness of a single electrode finger and its length is in the order of 1:29,500 while the ratio between the electrode width and length is approximately 1:650. Hence, the final FEM-based numerical model, built with COMSOL^®^ Multiphysics (Stockholm, Sweden) tools, is expected to require a huge number of elements in order to achieve accurate results. This causes long pre-processing, solving and post-processing periods in addition to the need for advanced computer hardware. To develop nevertheless a representative model that can be used for fast acquisition of results and multi-parametric simulations, various simplifications for the FEM model were considered.

As stated previously, the IDE design consists of 307 electrode pairs. Each set is connected to a sensor terminal representing the ground and voltage source, respectively. The terminal extends beyond the IDE structure to provide contact pads for the sensor reading device.

The design of the IDE sensors shows a clear redundancy in layout, hence a periodicity in the design is present that can be exploited. Consequently, simulation time and model complexity are reduced by modeling only one electrode pair, instead of simulating all 307 pairs. This simplification was carried out by applying periodic conditions to the external boundaries. Finally, the electrode length can be seen as an extension parameter of a 2D model to approximate the 3D case.

**Figure 5:**
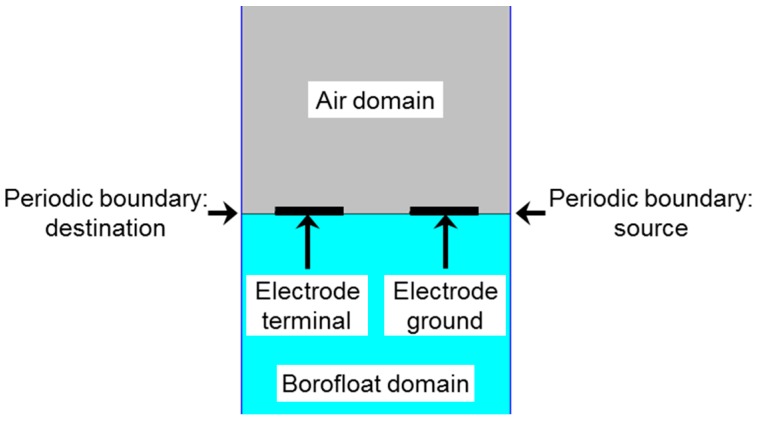
Simplified representative model of a blank sensor in 2D, showing an electrode finger pair surrounded by air and glass domains.

The 2D model used to simulate a blank IDE sensor is depicted in Figure 5. The model includes an electrode finger pair situated on top of a glass substrate, which is exposed to air. The FEM-based numerical model solves Gauss’s law for the electric field using the defined value of the electric potential as the independent variable. Therefore, the capacitance of the design can be calculated in the FEM model by two methods: calculation based on the terminal charge (Equation (3)), or calculation based on the surface integral of the stored electric energy *W*_e_ (Equation (4)):

(3) $$C=\frac{NQ}{2U_{0}}$$

(4) $$C=N\frac{2}{U_{0}^{2}}\int_{\Omega }W_{e}d\Omega$$

Here, *C* represents the capacitance of the system, *N* is the number of electrode fingers, *Q* is the total charge and *U*_0_ is the applied potential difference between terminal and ground electrode. Ω is the enclosed 2D model surface (domain).

The stored electric energy (*W*_e_) is calculated by Equation (5):

(5) $$W_{e}=\frac{1}{2}\varepsilon_{0}{\left(\underline{\varepsilon_{r}}\cdot E\right)}^{T}\cdot E$$

where ***ε_r_*** represents the relative permittivity tensor of the material under test and ***E*** the electric field vector. The sensor base materials used in this model are isotropic, which means that the relative permittivity is constant.

In the experimental work, an impedance analysis was performed to find the capacitive response of the IDE structure at different frequency values. Using the numerical model, a frequency analysis is also possible by applying parametric sweeps over a pre-defined frequency range in a frequency domain analysis. In principle, the frequency dependence of the material properties should be known in order to model the capacitance changes of the IDEs in a broader frequency range. The major contribution to the capacitance is related to the substrate material, which has the highest electric permittivity value. Unfortunately, information on the frequency-dependent electric permittivity of Borofloat glass is not provided by the manufacturer. Nevertheless, a constant value of ***ε****_r,glass_* = 4.6 at a frequency of 1 MHz was assumed and also used in the frequency analysis of the 2D model.

## 4. Results and Discussion

### 4.1. Impedimetric Characterization

In the following section, the acquired impedance and phase spectra plots (Bode plots) before and after the immobilization of spores and sterilization are presented and discussed. All measurements are carried out with air as surrounding sensor medium.

**Figure 6:**
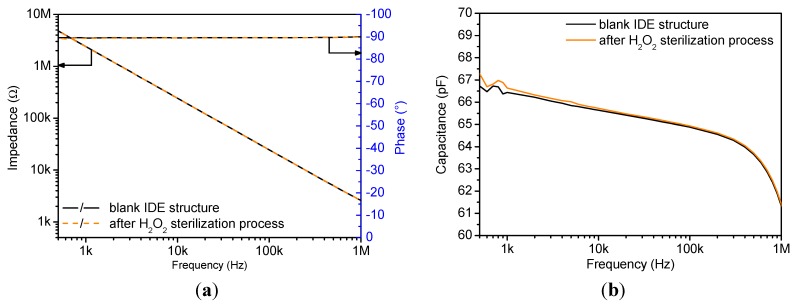
Analysis of a blank sensor exposed to the H_2_O_2_ sterilization process (**a**) Bode plot before and after sterilization; (**b**) plot of the corresponding capacitance spectra.

As stated in Section 2.1, a reference IDE is used to examine the impact of the sterilization process on the sensor set-up itself and to provide further information on the long-term behavior of the sensor output. The impedance of the sensor is derived from the ratio between the excitation voltage and the induced current, thus consisting of the impedance modulus (*Z*) and the phase shift (*φ*) between voltage and current. In Figure 6a, the Bode plots of the reference IDE before and after sterilization are depicted. As demonstrated in the figure, no changes of the impedance and phase are visible, which proves the stability and inertness of the sensor. In addition, the constant phase angle of about −90° indicates a capacitive behavior. This can be directly related to the non-conductive properties of the media surrounding the IDE. 

A detailed analysis of the IDE structure is then performed by applying Equation (6) to calculate the capacitance of the sensor:

(6) $$C=\frac{-\sin(\varphi )}{2\pi f\cdot Z}$$

This equation was developed using an equivalent circuit model consisting of resistive (R) and capacitive (C) elements, where *C* is the resulting capacitance, *φ* is the phase angle, *Z* the impedance taken from the Bode plot and *f* the respective frequency.

Figure 6b shows the plot of the calculated capacitance values derived from Figure 6a for a blank IDE structure prior and after sterilization. Both curves exhibit a frequency dependence of the capacitance in the range from 500 Hz to 1 MHz, which can be related to the properties of the sensor material (glass substrate, electrode structure and SU-8 walls). Furthermore, the capacitance plot shows minor deviations within the frequency range between 500 Hz and 5 kHz that can be related to the sterilization process. This implies the necessity of testing a differential sensor set-up for each experiment.

Further analyses of the blank IDEs were conducted to determine the base capacitance at a fixed frequency of 3 kHz, which shows minor fluctuation of ±0.9 pF around an average value of 66.1 pF. These small deviations might be explained by fluctuations in sensor production.

**Figure 7:**
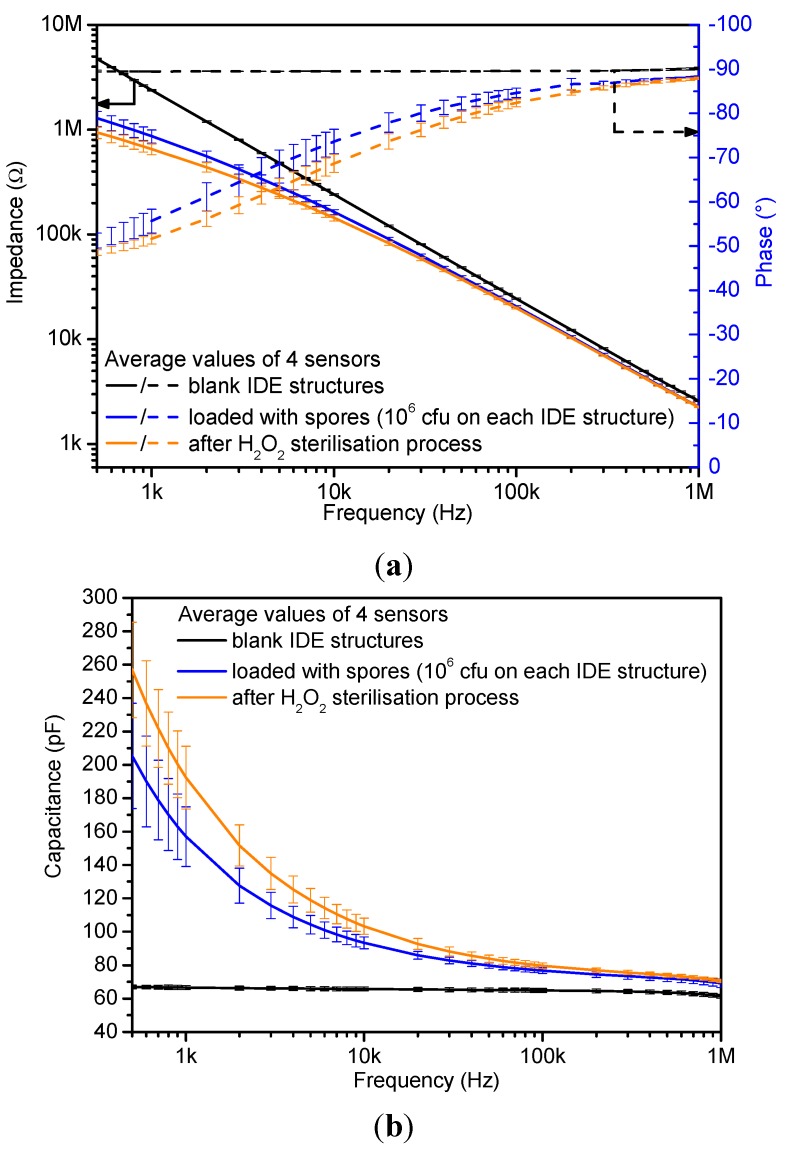
(**a**) Bode plots recorded with a blank IDE structure, after immobilization of *B. atrophaeus*-spores (10^6^ cfu on IDE structure) and after the H_2_O_2_ sterilization process. The impedance analyses were performed while the sensors were exposed to air; (**b**) plot of the derived capacitance spectra.

Figure 7a depicts the average impedance and phase spectra of four different IDE structures. In the first step, the blank structures were analyzed (black solid and dashed lines). The second analysis was conducted after the immobilization of spores onto the IDE (solid and dashed blue lines). The final analysis was performed after the H_2_O_2_ sterilization process (orange solid and dashed lines). The resulting Bode plots exhibit the impact of different test conditions on the IDEs. For example, the immobilization of microbiological spores results in a reduced impedance response in comparison to the empty IDE structure, which is related to the conductive properties of the microorganisms [16]. After the sterilization process, the impedance modulus further decreases as compared to the recorded spectrum of immobilized spores. The impedance change is related to the collapse and/or rupture of the spores, which was observed in previously published work [13]. The spore rupture is accompanied by a leakage of internal conductive substances [16,17]. Additional microbiological tests (count-reduction test) in parallel to IDE analyses have shown sample sterility. Since the capacitive behavior is still dominant, further analyses were performed using the capacitance spectra given in Figure 7b.

The study of the capacitance plot highlights that the sensor sensitivity is highest in the frequency range from 500 Hz to 10 kHz while it is reduced at frequencies higher than 10 kHz. However, as observed in Figure 6b, the frequency region to analyze the sensor response has to be restricted due to measurement instabilities between 500 Hz and 1 kHz. Therefore, the current and future sensor analyses are performed at a fixed frequency of 3 kHz. The resultant average capacitance of four tested sensors at this predetermined frequency and under different test conditions is noted in Table 1. The result demonstrates a distinguishable capacitance change of about 20 pF between the situation before and after the H_2_O_2_ sterilization. Finally, the one-point frequency analysis provides an extra advantage when e.g., designing a low-cost handheld read-out device.

**Table 1:** Summary of the one-point capacitance analyses of the three sensor states (average of 4 sensors) at a fixed frequency of 3 kHz.

Sensor State	C (*f* = 3 kHz) (pF)	Relative Error (%)
Blank sensors	66.1	2.6
Loaded with spores	115.7	7.2
After sterilization	135.0	4.3

### 4.2. Verification and Validation of the IDE Sensor

In parallel to the experiments in Section 4.1, analytical and numerical analyses of the blank IDEs were carried out. Table 2 summarizes the resultant capacitance values, where the results of the analytical expression and numerical simulations are in good agreement with each other. Therefore, Table 2 proves that the analytical expression defined in Equation (1) matches the values obtained by the numerical model. However, the relative error between experimental measurement and the numerical result of ideal electrodes amounts to about 12.4% (deviation between C_exp_ and C_ideal_ of Table 2).

**Table 2:** Summary of the capacitance values derived from the experiments and derived by analytical and numerical analyses.

IDE Geometry			Results			
			Experimental	Analytical (Equation (1))	FEM Model	
w (μm)	s (μm)	t (nm)	C_exp_ (pF)	C_anal_ (pF)	C_ideal_ (pF)	C_AFM_ (pF)
5	5	110	-	50.09	50.07	-
6	4	110	66.1	57.98	57.91	60.05
6	4	220	-	58.95	58.57	-

w: finger width; s: finger interspacing; t: finger thickness; C_ideal_: geometry as given in Figure 5; C_AFM_: IDE geometry based on AFM data.

**Figure 8:**
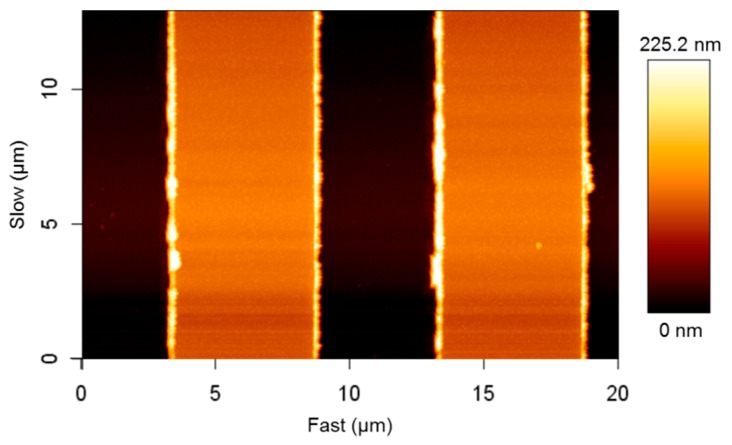
AFM characterization of the IDE structure depicting the uneven electrode edges due to the lift-off process. Scan directions of the AFM tip are given in fast (*x*-axis) and slow (*y*-axis).

Analysis of the IDE structure with atomic force microscopy (AFM) depicts uneven electrode edges, which are related to the lift-off process and the mechanical stability of the thin structures (Figure 8).

**Figure 9:**
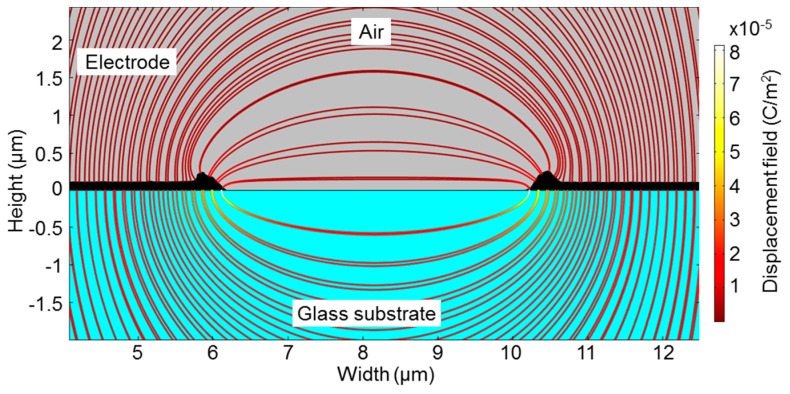
Distribution of the electric displacement field presented as streamlines between two electrode fingers. The electrode geometry determined by AFM characterization demonstrates a wide distribution of the fringing field and a reduced transverse field.

Hence, the model shown in Figure 5 was adapted with the AFM profile (as shown in Figure 9) and simulated. The result was an increase of the capacitance value in comparison to the previous value and thus, a decrease of the relative error to 9.15%. In addition to the error due to geometric uncertainty, air humidity, temperature and material purity have an effect on the capacitance measurements in contrast to the ideal conditions of the numerical model.

Furthermore, Table 2 shows that the height of the electrodes has a minor effect on the capacitance value as in the case of the blank sensor. The direct reason is related to the fact that, unlike parallel plate capacitors, the transverse fields have a smaller value (and hence effect) than fringing fields. This effect can be well perceived in Figure 9.

## 5. Conclusions and Outlook

In this work, a sensor consisting of interdigitated electrodes deposited on an insulating glass substrate was successfully introduced. A blank reference IDE structure was used to demonstrate the inertness and stability against the sterilization process using H_2_O_2_ vapor. Nonetheless, a reference electrode structure defined in a differential sensor set-up is still essential to capture possible variations such as process-induced changes of the sensor materials (electrode structure or SU-8-layer).

Resilient microorganisms (*B. atrophaeus*-spores), commonly applied for sterility tests in food industry, were immobilized on the sensing structure. A significant change in the impedance of the sensor was observed after spore immobilization. Whereby, after exposure to the sterilization process the spore morphology changed resulting in an impedance shift recorded by an impedance analyzer. Further evaluation of the data was performed calculating the capacitance of the IDEs using a simplified RC equivalent circuit.

From the output measurements an optimal fixed frequency of 3 kHz was determined for a latter impedance handheld device. As a result of the sterilization process, a capacitance change of about 20 pF was recorded at that fixed frequency. In addition, by means of analytical and numerical methods, a verification and validation of the IDE structure was performed. Analytical expressions provided in literature were analyzed and modified. For numerical validation a FEM-based simulation model of the IDEs was designed. Systematic simplification approaches of the model enabled the simulation of the complex geometry. Hence, further improvements and multiple tests can be achieved in short time and with the available hardware at hand. In future work, this IDE model will be applied to acquire further information about the electrical properties of the spores, such as electric permittivity and conductivity. Moreover, the validated FEM model can be applied to study different sensor modifications towards sensor optimization with regard to geometrical variations or spore deposition.
